# Sleep reactivation did not boost suppression-induced forgetting

**DOI:** 10.1038/s41598-020-80671-w

**Published:** 2021-01-14

**Authors:** Eitan Schechtman, Anna Lampe, Brianna J. Wilson, Eunbi Kwon, Michael C. Anderson, Ken A. Paller

**Affiliations:** 1 Department of Psychology, Northwestern University, Evanston, IL 60208 USA; 2 MRC Cognition and Brain Sciences Unit, University of Cambridge, Cambridge, CB2 7EF UK

**Keywords:** Cognitive neuroscience, Learning and memory, Forgetting, Sleep

## Abstract

Sleep's role in memory consolidation is widely acknowledged, but its role in weakening memories is still debated. Memory weakening is evolutionary beneficial and makes an integral contribution to cognition. We sought evidence on whether sleep-based memory reactivation can facilitate memory suppression. Participants learned pairs of associable words (e.g., DIET–CREAM) and were then exposed to hint words (e.g., DIET) and instructed to either recall (“think”) or suppress (“no-think”) the corresponding target words (e.g., CREAM). As expected, suppression impaired retention when tested immediately after a 90-min nap. To test if reactivation could selectively enhance memory suppression during sleep, we unobtrusively presented one of two sounds conveying suppression instructions during sleep, followed by hint words. Results showed that targeted memory reactivation did not enhance suppression-induced forgetting. Although not predicted, post-hoc analyses revealed that sleep cues strengthened memory, but only for suppressed pairs that were weakly encoded before sleep. The results leave open the question of whether memory suppression can be augmented during sleep, but suggest strategies for future studies manipulating memory suppression during sleep. Additionally, our findings support the notion that sleep reactivation is particularly beneficial for weakly encoded information, which may be prioritized for consolidation.

## Introduction

Sleep makes an integral contribution to memory by stabilizing previously acquired memories. Sleep-based consolidation supports declarative memories (concerning facts and events; e.g.,^1^), as well as nondeclarative memories (which include skill learning and implicit learning; e.g.,^2,3^). The active systems consolidation hypothesis suggests that declarative memories are selectively reactivated during non-rapid-eye-movement sleep (NREM) and that this process shapes and stabilizes cortical representations based on hippocampal–cortical interactions^4^.

By definition, sleep’s role in consolidation implies that it reduces forgetting. However, forgetting per se encompasses multiple processes, including memory-trace decay, proactive interference, and motivated memory suppression. Whereas sleep arguably rescues memory from deterioration, its role may be different with regard to memory control (the active, conscious control mechanisms that inhibit memories^5^). Humans can purposefully weaken specific memories via active suppression^6^, a mechanism which involves inhibition originating from the prefrontal cortex^7,8^. Like other forms of acquired inhibition such as extinction learning, memory control is inherently a form of learning and may therefore plausibly benefit from sleep. The neural trace supporting selective memory suppression, established during wake, may itself be reactivated and cemented during sleep, just as other nondeclarative products of learning (e.g., motor skills^3^, perceptual skills^2^) benefit from offline processing. Exploring the role of sleep in promoting memory control may prove useful in treating disorders such as post-traumatic stress disorder, which may be conceptualized as a memory control problem and is characterized by disrupted sleep and possibly suboptimal consolidation^5,9,10^.

The think-no-think paradigm (TNT^6^) has been used to study mechanisms of memory control. First, participants learn to associate a set of hint words (e.g., “Diet”) with target words (e.g., “Cream”). Next, they are required to repeatedly suppress some target words when exposed to the corresponding hint words (the *no-think* condition), while for a different set of pairs they repeatedly bring the associates to mind (the *think* condition). A third set of pairs are not presented during this stage, providing a baseline condition for comparisons. Studies have consistently shown that subsequently, when participants are asked to try to recall all pairs, no-think pairs are remembered less well than baseline pairs (i.e., suppression-induced forgetting), whereas think pairs are remembered better than baseline pairs^6^.

Studies exploring the long term effects of TNT manipulations have yielded conflicting results, with most reports suggesting that suppression-induced forgetting is diminished with time^11,12^, and only one showing long-lasting effects^13^. An outstanding question concerns the effect of offline consolidation periods, such as sleep, on memory suppression processes. Studies exploring the effects of sleep on motivated forgetting using other designs, such as directed forgetting, have yielded inconsistent results (see review in^14^). To date, three studies have directly focused on how sleep may impact the effects of the TNT manipulation on memory. Fischer and colleagues^15^ found no differential effect of memory control after a single night of sleep relative to a wake control condition. Conversely, when contrasting the effects of rapid-eye-movement (REM) sleep and slow-wave sleep on TNT in another experiment, they revealed that the former stage of sleep improved memory for suppressed items. The two other studies exploring sleep’s effect on memory control used daytime naps, which commonly include more SWS than REM. These studies supported the results obtained in the overnight study, and showed no differences between sleep and wake groups. Whereas Dehnavi and colleagues^16^ found that both groups showed worse memory for the no-think condition relative to the think condition following the delay, Davidson and colleagues^17^ found that suppression-induced forgetting was reduced after the delay, making it difficult to discern whether sleep had any substantial impact.

Results from sleep TNT studies suggest either that sleep does not spontaneously play a role in memory-control-related consolidation, or that consolidation of the inhibitory trace is overshadowed by consolidation of the to-be-suppressed memory trace itself. However, this does not necessarily mean that sleep cannot be harnessed to selectively enhance suppression by employing techniques to bias memory reactivation. If successful, such procedures may reveal that inhibitory traces benefit from sleep under certain circumstances, and may also lead to novel procedures useful in clinical settings to improve well-being^10^. We hypothesized that sleep’s potential for impacting memory control may become apparent if memory reactivation during sleep is manipulated to suppress memories, tilting the scale toward suppression-specific consolidation. To test this idea, we selectively targeted the reactivation of no-think pairs using targeted memory reactivation (TMR). TMR is a technique involving the unobtrusive presentation of learning-related cues during sleep, thereby impacting memory consolidation^18^. This technique has repeatedly been shown to enhance prior learning^19^, and has also been used to weaken specific memories under some circumstances^20,21^. Importantly, a recent study showed that TNT-induced memory suppression can be achieved using unconsciously presented cues during wake, indirectly supporting our assumption that targeting suppression-related memories during sleep may selectively impact retrieval^22^.

**Figure 1:**
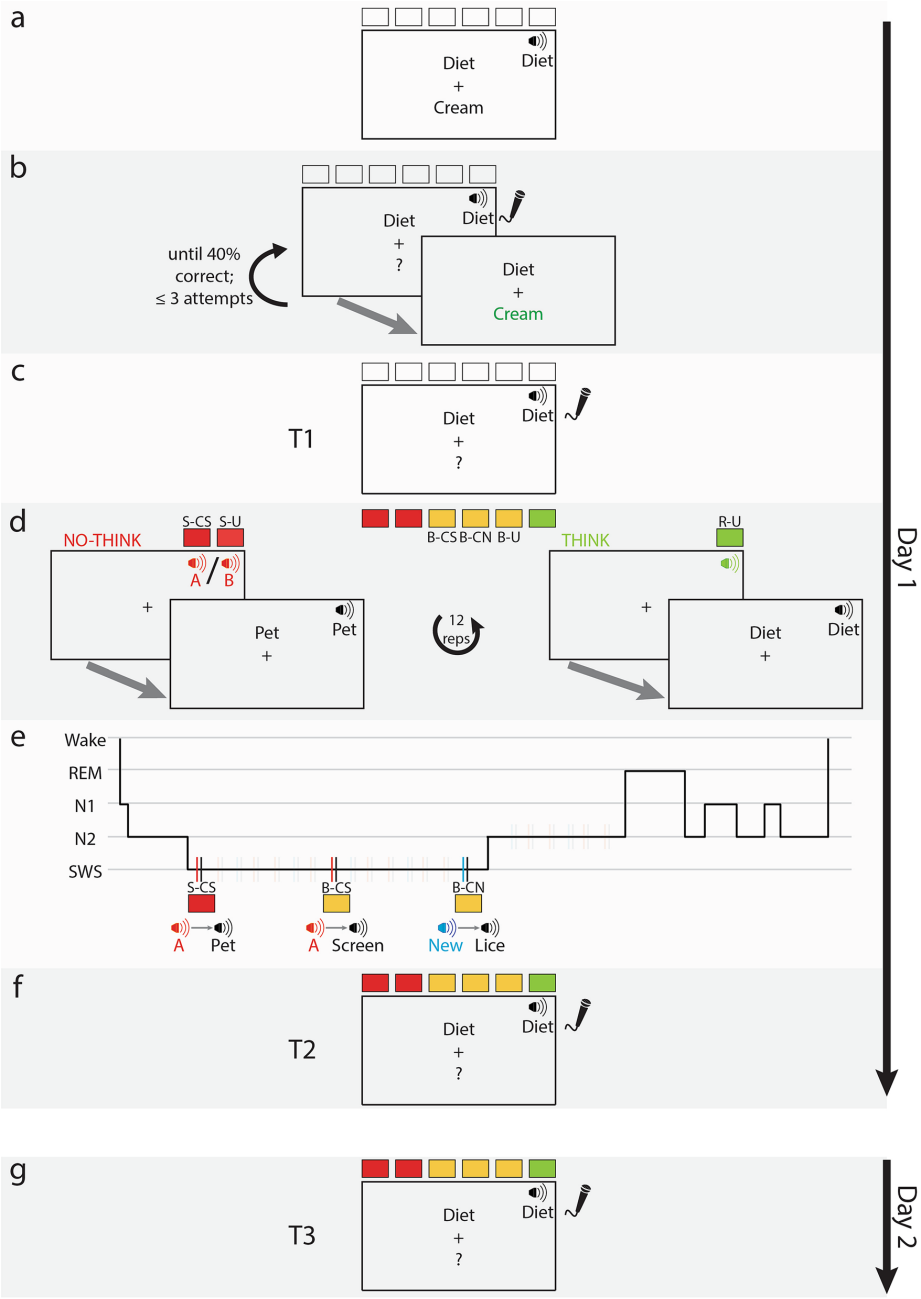
Outline of the experimental procedure. (**a**) Participants were first passively exposed to a set of word-pairs, while simultaneously hearing the hint word (e.g., “Diet”). (**b**) Next, participants were presented with the hint word visually and through headphones and asked to respond into a microphone with the appropriate target word (e.g., “Cream”). Responses were immediately followed by visual feedback. If participants did not correctly respond to at least 40% of the pairs, this stage was repeated until the criterion was reached or three unsuccessful attempts were made. (**c**) Participants were then tested on all pairs without feedback (T1). The hint words were identical to those in the previous stage, but no feedback was provided. (**d**) The pairs were then divided into 6 groups of 12 pairs each (see Table 1). Participants next initiated the think-no-think stage. They first learned to associate abstract instructional sounds with think and no-think instructions, practiced the task, and then started the six-block protocol. When hint words were preceded by instructional no-think sounds (red, left), the participants had to avoid thinking of the appropriate target word. Two 12-pair sets (S-CS, S-U) were used for this condition, each consistently preceded by one of two sounds (termed sounds A and B, respectively, in this figure). When hint words were preceded by instructional think sounds (green, right), the participants had to try and think of the appropriate target word. One 12-pair set (R-U) was used for this condition. Altogether, each pair was presented 12 times during this stage. Three sets of words, termed baseline sets (B-CS, B-CN, B-U), were not presented during this stage. (**e**) During NREM stages of a subsequent nap, sounds belonging to three conditions were repeatedly presented. All 12 hint words from the S-CS condition were presented, preceded by the associated instructional no-think sound (sound A); all 12 hint words from the B-CS condition were presented, preceded by the same instructional no-think sound (sound A); all 12 hint words from the B-CN conditions were presented, preceded by a novel sound not associated with any instructions. (**f**) Following the nap, participants were again tested on all word-pairs and instructed to try their best, regardless of previous instructions. T2 was, therefore, identical to T1. Participants were then dismissed. (**g**) On the next day, participants returned to complete another identical test (T3).

Participants in our experiment initially learned word-pairs, and then completed the TNT stage, during which instructions to suppress or recall the target words were conveyed using sounds (Fig. 1). Next, participants slept with polysomnographic monitoring, including electroencephalography (EEG), electrooculography, and electromyography, and were exposed to one of two sounds conveying no-think instructions along with hint words of suppressed pairs. We hypothesized that the presentation of suppression cues during sleep would enhance the suppression effect established during wake. In addition to these stimuli, we also presented the same suppression sound followed by hint words from the baseline condition. This manipulation was designed to check whether memories that were not suppressed during wake can be weakened by pairing them with suppression instructions during sleep. Retrieval for all sets was tested after the nap and again the next day. Results showed that TMR did not reduce memory for the cued no-think pairs, nor did it affect memory for baseline pairs presented during sleep.

## Materials and methods

### Participants

Participants were native English-speakers who did not suffer from any neurological or sleep disorder and claimed to be able to nap in the afternoon. They were asked to go to bed later than usual the night before the study, wake up earlier than usual in the morning, and avoid caffeine on the first day of the study. Data were collected from 54 participants, but data from 23 participants were excluded from analysis: 14 participants were not sufficiently cued during NREM sleep (see details below); 5 participants were not able to complete learning requirements (see details below); 2 participants withdrew from the study before the nap portion of the study; and 2 participants could not complete the protocol because of technical issues and were dismissed before the nap portion of the study. The final sample included 31 participants (26 women and 5 men) with ages ranging between 18 and 29 years (mean ± SD = 20.94 ± 2.79). The Northwestern University Institutional Review Board approved the procedure. The experiment was performed in accordance with relevant guidelines and regulations. Participants were paid US$10 per hour for their efforts.

### Materials

Stimuli consisted of 86 associable word-pairs (e.g., Diet–Cream), including six sets of 12 pairs each (test sets) and 14 pairs that were not used for memory testing (filler sets). All word-pairs are presented in Supplementary Table 1. These pairs were not tightly associated, nor were they randomly linked. Instead, the pairs were chosen to share some connection, enabling participants to easily form links between pairs during learning. By choosing association strengths which lie in the middle of the range, as in previous studies^6,7^, we hoped to avoid both ceiling and floor effects in recall performance. Test sets were each assigned to one of six different conditions for each subject (see below). Filler sets were used for several purposes: (a) training on think and no-think instructions; (b) avoiding the serial-position effects in learning blocks and the warm-up effect in the first trials of testing blocks; and (c) reinstating the original study-phase context (see below). Sound files for all 86 hint words were recorded by a female experimenter (mean duration: 480 ± 77 ms, range 349–599 ms). Additionally, five 1-s sounds were used to convey TNT instructions. Sounds were not associated with any of the hint or target words (a sword swing sound effect, a computer-generated beep sequence, etc.). Visual stimuli were presented on a 1920 × 1080 pixel screen (P2417Hb, Dell Inc.) and words were presented at a visual angle of approximately 12°–20°. During wake portions of the study, sounds were delivered using headphones (MDR-7502, Sony Inc.) and spoken responses were recorded using a microphone (FV-5, Sony Inc.). During sleep, sounds were delivered using speakers (AX-210, Dell Inc.). Stimulus presentation was controlled by Neurobehavioral Systems Presentation (v18.3 for the wake part of the task and v17.2 for the sleep part of the task), which also recorded audio files and estimated response times online. Scripts and stimuli are available upon request.

### Word-pair learning procedure

Participants arrived at the lab between 11:30 am and 1:00 pm. Informed consent was obtained from all participants. They then filled out a demographic questionnaire. An attention test was administered to confirm participants were attentive and able to respond quickly to stimuli. In this task, termed the *red-square task*, a red square shifted between left and right positions every 100 ms for a random period of time (range 1–3 s), then remained static at one of the two locations for 450 ms, and then resumed shifting. Participants were required to click the correct mouse button (left or right) within 450 ms during each static period. The task ended when the participant responded correctly for eight out of ten consecutive trials.

Participants next started the TNT task (Fig. 1). Throughout this task, the participant sat in the same room as the experimenter. An instruction sheet was handed to the participant at the beginning of each phase. First, each of 86 word-pairs were presented on the screen, one at a time, along with a single auditory presentation of the spoken hint word (Fig. 1a). Words appeared visually for 2000 ms, followed by a 1000-ms inter-trial interval (ITI). Pair order was pseudorandomized and identical for all participants for this and all subsequent stages of the study. Next, a microphone was placed in front of the participant. Each of the 86 hint words were then presented one at a time, simultaneously on the screen and through the headphones. The participant had 4000 ms to respond by speaking the target word (Fig. 1b). Regardless of whether the response was correct or not, the correct target word was presented, followed by a 1300 ms ITI beginning 500 ms after feedback onset. Responses were scored online by the experimenter. To continue to the next stage, the participant had to be correct for at least 40% of the words. If this criterion was not reached, this stage was repeated. If the participant did not reach criterion after three attempts, they were paid for their time and dismissed (*n* = 4).

Next, participants were tested on their memory of all 86 word-pairs without feedback (T1; Fig. 1c). Hint words were identical to hint words used during training and the participant had to produce the target word. Details followed that of the previous stage, except that no feedback was provided and the ITI was 2300 ms. The microphone was removed immediately after T1.

In the next stage (Fig. 1d), each of the six word-pair groups was automatically assigned to one of the six conditions (Table 1). Three of the sets were designated to undergo the TNT manipulation, including two no-think sets and one think set. The other three sets did not undergo the TNT manipulation (i.e., baseline sets). The first letter in the acronym for each condition signifies the manipulation it included: either the no-think manipulation (S; Suppression), the think manipulation (R; Response), or neither (B; Baseline). The letters after the dash describe the subsequent manipulation during sleep (see Table 1). The assignment of sets to these six conditions was pseudorandomly determined for each participant using an algorithm that assigned conditions in a manner minimizing the variability in T1 accuracy rates and response times (RTs) within each triplet of conditions (i.e., the TNT sets: S-CS, S-U, R-U; and the baseline sets: B-CS, B-CN, B-U). Briefly, the algorithm considered all the combinations of three groups out of six ($\left(\genfrac{}{}{0pt}{}{6}{3}\right)$ = 20) and found the combination that minimized the variability in T1 accuracy rates within each triplet (i.e., the three TNT sets and the three baselines sets). If more than one combination reached the minimum, the same algorithm was used to minimize T1 RT variability. Each of the two chosen triplets was then randomly assigned to be either a TNT or a baseline triplet. Finally, within each triplet, the algorithm randomly assigned each of the three conditions.

**Table 1:** Within-subject experimental conditions.

Condition	Acronym	TNT procedure	Cuing during sleep
Suppression–cued suppression	S-CS	No-think	Suppression sound
Suppression–uncued	S-U	No-think	None
Response–uncued	R-U	Think	None
Baseline–cued suppression	B-CS	None	Suppression sound
Baseline–cued novel	B-CN	None	Novel sound
Baseline–uncued	B-U	None	None

### Think-no-think manipulation

For the TNT manipulations, participants had to learn the instructions and learn to associate the different instructional sounds with each set of instructions. There were two suppression cues and two response cues, which were randomly assigned for each participant out of a set of five sounds. The two suppression cues and one of the response cues were each consistently associated with a specific set of words (i.e., there were three specific sounds associated with each of the following conditions: S-CS, S-U, and R-U). The additional response cue was included for symmetry and not used with any word-pair group. To learn which sounds conveyed which instructions, participants were presented with a screen and clicked on specific boxes to hear the sounds associated with think and no-think instructions. After learning these associations, they were tested using a two-alternative-forced-choice test to see whether they knew which instructions were conveyed by each sound. If they made any errors, they were asked to learn the sounds again. This was repeated until they made no errors.

The TNT manipulation was introduced to the participants as a task intended to measure their ability to pay attention and ignore distractions. However, we did not introduce a cover story to directly mislead participants into thinking that no memory test would be given later for the suppressed sets. TNT trials all followed the same general structure (Fig. 1d): first, a 1-s instructional TNT sound cue was presented followed immediately by a hint word from the appropriate set. The hint word was presented on screen and through headphones. Participants had to either keep the target word out of mind (no-think) or try to recall it (think), according to the instructional TNT sound that was played. Either way, no response was required. Participants were explicitly instructed not to substitute the no-think target word with a new, unrelated word, but rather to only suppress the target word and keep it out of mind^23^. The hint words disappeared from the screen after 4000 ms, followed by a 400-ms ITI.

To confirm that the instructions were understood, a 24-trial practice block, including eight of the filler words (each presented three times), was initiated. Participants then filled out a questionnaire assessing their understanding of the instructions, which was followed by spoken clarification if necessary (Supplementary Table 2). Then, another practice block ensued, which was identical to the previous practice block and followed by a 5-min break. After returning, participants had a chance to refresh their memories on the sound-instruction associations and were tested on these again, using the aforementioned protocol. Finally, participants started the TNT stage described above, which was divided into six blocks with a 1-min inter-block interval. Each of the 36 hint words (i.e., all words from the S-CS, S-U and R-U conditions) was presented twice per block (12 times total). Along with eight filler words, each block therefore consisted of 80 trials altogether. After three of the six blocks, participants were questioned again to assess their understanding of the task and clarifications were provided if necessary (Supplementary Table 2). When this stage was over, participants were given a surprise test on the sound-instruction associations. One participant who failed this test was dismissed from the study at this stage.

### Targeted memory reactivation

Five min after completion of the TNT stage, participants were fitted with an EEG cap, entered the sleep chamber, and were allowed to nap for 90 min (Fig. 1e; Table 2). Altogether, the break and the polysomnography equipment set-up, up until when the lights were turned off, lasted 19.35 ± 0.62 (SEM) minutes. Lights were turned off and white noise was presented throughout the nap. During the NREM stages of the nap, sounds were unobtrusively presented. The letters after the dash in each condition’s acronym (see Table 1) represented details on sleep-related reactivation: three word-pair groups were not cued during sleep (uncued; U), two other groups were cued using a suppression sound as described below (cued suppression—CS) and another group was cued using a novel sound (cued novel—CN). Stimuli belonging to the three cued groups were presented in a pseudorandom order: (a) one of the two suppression sounds (i.e., the one associated with condition S-CS; Sound A in Fig. 1d,e), followed immediately by a S-CS hint word; (b) the same sound followed immediately by a B-CS hint word; (c) a novel sound, that was not presented during wake, followed immediately by a B-CN hint word. This last condition was used to determine whether the hypothesized detrimental effect expected for the B-CS set was causally related to the coupling of these baseline pairs with the no-think sound, or more generalized such that it was evident for the B-CN condition as well. In total, 36 sound-word conjunctions were repeatedly presented to each participant. Stimulation was halted upon any sign of arousal and resumed when participants reached NREM. Each word began immediately when the prior sound terminated (i.e., the onset-to-onset interval was 1 s, which was the duration of the suppression and novel sounds). The offset-to-onset interval between sound-word conjunctions was randomly chosen to be 7, 8, or 9 s. Each run through all 36 stimuli therefore lasted approximately 5 min. Participants who were not exposed at least once to a minimum of 30 of the 36 stimuli during NREM stages were excluded from analysis. For two participants who received fewer than 36 stimuli, data from those that were not presented during NREM were omitted from analysis.

**Table 2:** Sleep architecture and cuing during sleep.

	Mean	SEM	%
**Architecture**			
Wake (min)	12.84	1.1	13.36
N1 (min)	10.02	1.43	10.42
N2 (min)	28.48	2.78	29.64
N3 (min)	39.71	3.57	41.32
REM (min)	5.06	1.04	5.27
Total sleep time (min)	83.27	1.42	(86.64)
**Number of cues in**			
N2	22.71	5.13	12.99
N3	150.13	18.15	85.88
Other stages	1.97	0.44	1.13
**Number of cues during NREM per set**			
S-CS	57.23	5.15	33.11
B-CS	57.84	5.13	33.46
B-CN	57.77	5.23	33.43

### Post-sleep memory tests

Participants were woken up after approximately 90 min, and were then allowed to wash off any remaining electrolyte following removal of recording electrodes. The task was resumed at least 10 min after the nap was terminated. Participants first repeated the previously described red-square task designed to confirm that participants are able to respond quickly to presented stimuli. We thus inferred that responses were not grossly distorted by sleep inertia (the acute state of lowered arousal immediately after awakening^24^). Then, the microphone was reintroduced and participants were tested on all word-pairs and asked to do their best regardless of previous instructions to suppress (T2; Fig. 1f). The first six trials of this stage consisted of the six filler words that were not used for TNT training. This arrangement was meant to reinstate the context of the learning stages, during which these words were last presented (i.e., as opposed to the TNT context). The parameters used for this test were identical to those used in T1. After completing this stage, participants were asked not to rehearse the words before the next test, scheduled for the next day. Participants were also asked whether they heard any sounds during sleep, and those who did were presented with the novel sound and all TNT sounds and asked for each whether they remember hearing it during sleep. Finally, participants were paid.

The following day, participants came back to the lab. They first completed the red-square task, and then a memory test for word-pairs identical to T2 (T3; Fig. 1g). They then completed several questionnaires intended to reveal their strategy while completing the task. The results of these questionnaires were not used in our analyses, but are presented in Supplementary Table 3. Finally, participants were paid for their participation in the second day's session and were dismissed. T3 data and responses to the questionnaires are missing for one participant, who withdrew from the study before this stage.

### Electroencephalography and polysomnography

EEG was recorded using a 32-channel system of Ag/AgCl active electrodes (Biosemi ActiveTwo, Amsterdam). In addition to the scalp electrodes, contacts were placed on the mastoids, next to the eyes, and on the chin. All recordings were made at 512 Hz. Sleep scoring based on the guidelines of the American Academy of Sleep Medicine^25^ was done online (while the participant was sleeping, for the purpose of controlling TMR cues) and, more formally, offline using EEGLAB^26^ and sleepSMG (http://sleepsmg.sourceforge.net) packages for Matlab 2016b (MathWorks Inc., Natick, MA). For offline scoring, the electrophysiological data were re-referenced to the averaged mastoids and filtered using a two-way least-squares FIR bandpass filter between 0.4 Hz and 60 Hz (*pop_eegfilt* function in EEGLAB). Noisy channels were replaced with interpolated data from neighboring electrodes using the spherical interpolation method in EEGLAB. Offline scoring was done by two independent raters not informed about when sounds were presented.

### Analysis

All analyses were completed using Matlab 2016b (MathWorks Inc., Natick, MA). Recall accuracy was assessed by the experimenter immediately after the participants' responses on a trial-by-trial basis. This scoring was confirmed and corrected by conducting offline analyses of the recorded audio files. RTs (i.e., the time interval between the presentation of the hint word and the triggering of the microphone) were also collected online per trial by Neurobehavioral Systems Presentation (v18.3). This analysis considered correct trials only, because most incorrect trials were omissions that did not include any spoken response. Correct trials in which the participant’s first utterance was not a word (e.g., pre-word fillers such as “Um”) were omitted from RT analysis. In total, 1.22% of correct trials were omitted, and they were equally distributed between the six experimental conditions (see Table 1) in all three tests (T1, T2, and T3; χ^2^ = 3.14, *p* = 0.68 collapsed over tests, *p* > 0.31 for each individual test). It is therefore unlikely that omitting these trials, which possibly reflected worse memory for certain word-pairs, had a major impact on our analyses of the differences between conditions.

Statistical analyses were conducted using linear mixed-effects models (*fitlme* function in Matlab), containing a random effect of participants. This method is ideally suited for within-subject analyses because it takes into account the hierarchal structure of the data (e.g., trials nested within participants) and accounts for differences in the number of trials for different conditions and participants. The fixed effects of different measures were calculated in different tests (e.g., condition, recall in T1). All tests were two-tailed. *F*-test and *t-*tests were used to analyze fixed effects (using the *anova* function for the *LinearMixedModel* class and the outputs of the *fitlme* function in Matlab).

## Results

### After learning was accomplished, recall was assessed in the initial test (T1)

Participants first attempted to learn 86 associable word-pairs and then recall at least 40% of the target words correctly when given the corresponding hint word (Fig. 1b). Up to three attempts were allowed to reach this recall criterion. The mean number of attempts needed was 1.65 ± 0.14 (SEM). For 52% of the participants (16/31), the criterion was reached on the first attempt. In the last phase of training, the average percentage of words correctly recalled was 57.8% ± 1.6% (SEM).

Training was followed by a test with no feedback (Fig. 1c). Average accuracy in this test (T1) was 82.14 ± 1.5% (SEM) and the average time to recall the correct word was 1415 ms ± 28 (SEM). The three conditions that would later undergo the TNT manipulation (S-CS, S-U, R-U) were not different for either accuracy or RT (*F*(2,1109) = 0.35, *p* = 0.7 and *F*(2,888) = 0.37, *p* = 0.69, respectively; Table 3). Similarly, the three baseline conditions (B-CS, B-CN, B-U) were not different for either accuracy or RT (*F*(2,1109) = 0.85, *p* = 0.43 and *F*(2,949) = 2.24, *p* = 0.11, respectively).

**Table 3:** T1 results.

Condition	Recall rates (%) ± SEM	Response times (ms) ± SEM
S-CS	77.2 ± 0.03	1440 ± 47
S-U	77.1 ± 0.02	1478 ± 47
R-U	79.3 ± 0.02	1471 ± 39
B-CS	85.1 ± 0.02	1435 ± 44
B-CN	85.4 ± 0.02	1423 ± 51
B-U	82.3 ± 0.03	1516 ± 56

Our analytic approach throughout this paper considers absolute changes in accuracy levels (e.g., T1 accuracy subtracted from T2 accuracy), and does not correct for performance in T1. Analyses based on proportional scores (e.g., T2 accuracy divided by T1 accuracy), which produced qualitatively similar results for key comparisons, are presented in the Supplementary Materials.

### Think-no-think procedures altered later memory retrieval

During the TNT stage (Fig. 1d), participants were presented with hint words and required to either suppress memory of the appropriate target word (for words belonging to the S-CS and S-U sets) or recall the appropriate target word (for words belonging to the R-U set). Which response was required, suppress or recall, was conveyed using instructional sounds that preceded hint-word presentations.

As a manipulation check, we compared the three conditions that were not cued during sleep, which consisted of a no-think set (S-U), a baseline set (B-U), and a think set (R-U). The standard way of analyzing TNT data excludes all pairs that were not correctly remembered in T1, before the TNT manipulation commenced. The dataset comprised of all remaining pairs is termed the *conditionalized* dataset^7,27^. One limitation of using this dataset is that memory gains (i.e., pairs not recalled in T1 but recalled in later tests), which are common in sleep^28^, may go unacknowledged. We therefore conducted our analyses twice, once using the complete dataset and once using the conditionalized dataset, to allow comparison with the previous literature.

Using the complete dataset, we first considered the three conditions using two accuracy change scores, T1 versus T2 (the test taken immediately after the nap) and T1 versus T3 (the test taken the next day). We found a main effect of condition (*F*(2,2190) = 4.34, *p* < 0.05) and of delay (*F*(1,2190) = 4.57, *p* < 0.05), but no interaction (*F*(2,2190) = 1.66, *p* = 0.18). Compared to results at T1, overall accuracy improved less at T2 than at T3 (4.1% difference in performance). To follow up the main effect of condition, we found that accuracy was significantly higher for the R-U condition relative to the S-U condition (*t*(2190) = 2.93, *p* < 0.01; 6.5% difference) and there was a trend towards higher accuracy for the R-U condition relative to the B-U condition as well (*t*(2190) = 1.85, *p* = 0.06; 4.3% difference). S-U and B-U conditions were not significantly different (*t*(2190) = 1, *p* = 0.32). Overall, this analysis indicated that the TNT manipulation significantly affected memory, as reflected by accuracy rates. Importantly, however, there was no significant suppression-induced forgetting (i.e., memory for the S-U pairs was not worse than for the B-U pairs) in this analysis. Additionally, it showed that accuracy levels were higher in T3 relative to T2 (as reflected by the main effect of delay).

**Figure 2:**
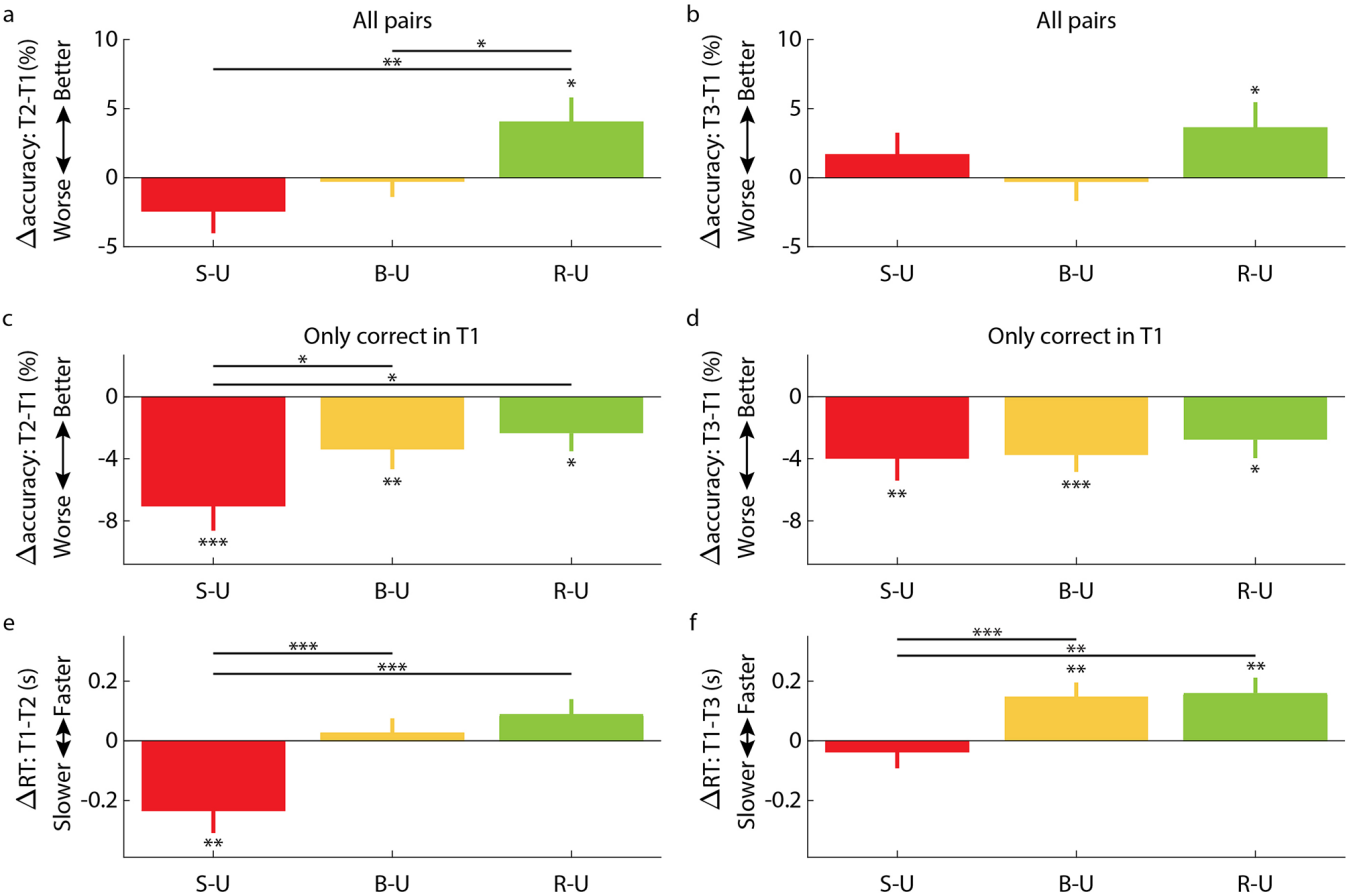
No-think and think manipulations effectively influenced memory retrieval. (**a**) Change in recall accuracy from T1 (pre-sleep) to T2 (immediately after sleep) for the three conditions with no corresponding cues during sleep (i.e., a suppression condition, a baseline condition, and a response condition). (**b**) Change in recall accuracy from T1 (pre-sleep) to T3 (the next day) for the three conditions with no corresponding cues during sleep. (**c**,**d**) These analyses repeated considering only pairs that were correctly recalled in T1 (i.e., the conditionalized dataset). (**e**) Change in RT from T1 to T2 for the same three conditions. (**f**) Change in RT from T1 to T3 for the same three conditions. Values reflect coefficient estimates produced by the linear model; error bars denote standard error of the mean for coefficients. *p < 0.05; **p < 0.01; ***p < 0.001. *S-U* suppression–uncued, *B-U* baseline–uncued, *R-U* response–uncued.

Although there was no indication that the TNT manipulation differentially impacted the results of T2 and T3 (i.e., the interaction term was not significant), we conducted separate planned analyses of forgetting across the two delays (Fig. 2a,b). Considering the change in accuracy from T1 to T2, we found a significant difference between conditions (*F*(2,1112) = 5.07, *p* < 0.01; Fig. 2a). The increase in accuracy was higher for the R-U condition relative to both the B-U and S-U conditions (*t*(1112) = 2.01, *p* < 0.05; *t*(1112) = 3.16, *p* < 0.01, respectively), which were not significantly different from each other (*t*(1112) = 1.07, *p* = 0.29). Out of the three conditions, only in R-U was the change in accuracy significantly different from zero (*t*(1112) = 2.32, *p* < 0.05; *p* > 0.12 for the other conditions). For the change in accuracy from T1 to T3, results did not differ across TNT conditions (*F*(2,1075) = 1.34, *p* = 0.26; *p* > 0.1 for all pairwise comparisons; Fig. 2b). Again, R-U was the only condition for which the change in accuracy was significantly different from zero (*t*(1075) = 1.96, *p* < 0.05; *p* > 0.29 for the other conditions).

A standard way of analyzing think-no-think data is to focus only on the conditionalized dataset^7,27^. We therefore repeated our analyses using pairs that were correct in T1. We first considered both T2 and T3. Our analysis revealed a main effect of test time (*F*(1,1739) = 3.86, *p* < 0.05), with more benefits observed in T3 relative to T2. Next, we observed a main effect of word-pair group (*F*(2,1739) = 3.3, *p* < 0.05). As with the complete dataset, the benefit in accuracy for the conditionalized dataset was significantly lower for the S-U condition relative to the R-U condition (*t*(1739) = 2.5, *p* < 0.05). Importantly, in this dataset the benefit for the S-U condition was also lower relative to the B-U condition (*t*(1739) = 2.02, *p* < 0.05), replicating prior work on suppression-induced forgetting. The R-U and B-U conditions were not significantly different (*t*(1739) = 0.69, *p* = 0.49). Like before, we did not observe an interaction between test time and condition (*F*(2,1739) = 1.67, *p* = 0.19), which, if significant, may have indicated that our manipulation differentially affected T2 or T3. Nonetheless, we conducted planned analyses for T2 and T3 separately. We found a significant difference among conditions when considering T1 and T2 (*F*(2,884) = 3.5, *p* < 0.05; Fig. 2c), but not when considering T1 and T3 (*F*(2,855) = 0.37, *p* = 0.69; Fig. 2d). The former effect was explained by an accuracy reduction in the S-U condition relative to both B-U and R-U conditions (*t*(884) = 2.12, *p* < 0.05 and *t*(884) = 2.53, *p* < 0.05, respectively), showing suppression-induced forgetting. B-U and R-U conditions were not significantly different in T2 (*t*(884) = 0.64, *p* = 0.52). Relative to T1, accuracy got worse for all three condition in both T2 (*t*(884) = -4.3, *p* < 0.001, *t*(884) =− 2.7, *p* < 0.01, *t*(884) =− 2.02, *p* < 0.05 for S-U, B-U and R-U, respectively) and T3 (*t*(855) =− 2.88, *p* < 0.01, *t*(855) =− 3.49, *p* < 0.001, *t*(855) =− 2.31, *p* < 0.05 for S-U, B-U and R-U, respectively).

Accuracy levels reflect a binary measure for each word (recalled or not) and may therefore fail to capture more nuanced effects on memory retrieval, such as ease of recall. We therefore considered the effects of our manipulation on RTs in the conditionalized set. Only RTs of correct trials were considered for these analyses. Considering the changes in RT between T1 and subsequent tests (T2 vs. T3) for different word-pair groups (S-U, B-U, and R-U), we found a main effect of delay (*F*(1,1620) = 10, *p* < 0.01), with faster responses observed in T3 relative to T2. Additionally, we found a main effect of TNT condition (*F*(2,1620) = 9.56, *p* < 0.001). RTs were significantly slower for the S-U condition relative to the R-U condition (*t*(1620) = 4.08, *p* < 0.001) and relative to the B-U condition (*t*(1620) = 3.92, *p* < 0.001). The R-U and B-U conditions were not significantly different (*t*(1620) = 1.09, *p* = 0.28). As with accuracy benefits, there was no interaction between delay and TNT condition (*F*(2,1620) = 1.42, *p* = 0.24). This shows that RTs were faster on T3 relative to T2, but these differences were not modulated by condition.

Analyzing T1-to-T2 changes, we found a main effect of TNT condition (*F*(2,818) = 11.84, *p* < 0.001; Fig. 2e). Recall was slower in the S-U condition than in both the B-U condition (*t*(818) = 4.41, *p* < 0.001) and the R-U condition (*t*(818) = 4.56, *p* < 0.001). The B-U and R-U were not significantly different (*t*(818) = 1.24, *p* = 0.22). The only condition for which the change in RT was significantly different from zero was S-U (*t*(818) = -3.14, *p* < 0.01; *p* > 0.07 for the other conditions).

Unlike the nonsignificant results observed for accuracy rates, the RT results analyzed for T3 alone revealed a significant effect of the TNT manipulation (*F*(2,808) = 7.11, *p* < 0.001; Fig. 2f). Responses for the S-U condition were slower relative to both the B-U condition (*t*(808) = 3.36, *p* < 0.001) and the R-U condition (*t*(808) = 3.17, *p* < 0.01), demonstrating a suppression-induced forgetting effect. The B-U and R-U conditions were not significantly different (*t*(808) = 0.2, *p* = 0.84). For both the B-U and R-U conditions, RTs were significantly faster at T3 relative to T1 (*t*(808) = 3.14, *p* < 0.01, *t*(808) = 3.11, *p* < 0.01, respectively; *p* = 0.49 for S-U).

Finally, we considered whether differences between participants in the percentage of time spent in a specific sleep stage predicted their change in performance over the nap (i.e., at T2 relative to T1). The percentage of time spent in stage 3 of sleep (N3, also known as slow-wave sleep) did not significantly correlate with changes in RTs or accuracy levels for either the S-U or R-U conditions (*p* > 0.14, uncorrected). Similarly, the percentage of time spent in Non-REM sleep (including stages 2 and 3 of sleep) and in REM sleep did not correlate with any behavioral measures for these two conditions (*p* > 0.08, uncorrected).

Taken together, differential patterns of memory performance due to the TNT manipulation were evident when comparing T1 results versus T2 results (i.e., after learning versus after both the TNT procedure and the nap). Given that effects of the manipulation were generally reduced on the subsequent day, T3 results are not included in subsequent analyses.

### No evidence that TMR enhanced suppression

We next considered the effects of TMR on memory effects produced by the TNT procedure. Two main hypotheses were tested: (a) whether TMR can further enhance memory-suppression that was established via no-think instructions during wake (e.g.,^21^); and (b) whether memory suppression could be applied de-novo during sleep to impair subsequent retrieval (e.g.,^20^). Testing these hypotheses made use of the three word-sets used for cuing during NREM sleep. For these conditions, hint words were presented during sleep, preceded by either a sound conveying suppression instructions (S-CS and B-CS conditions) or a novel sound that was not presented during wake (B-CN condition).

**Figure 3:**
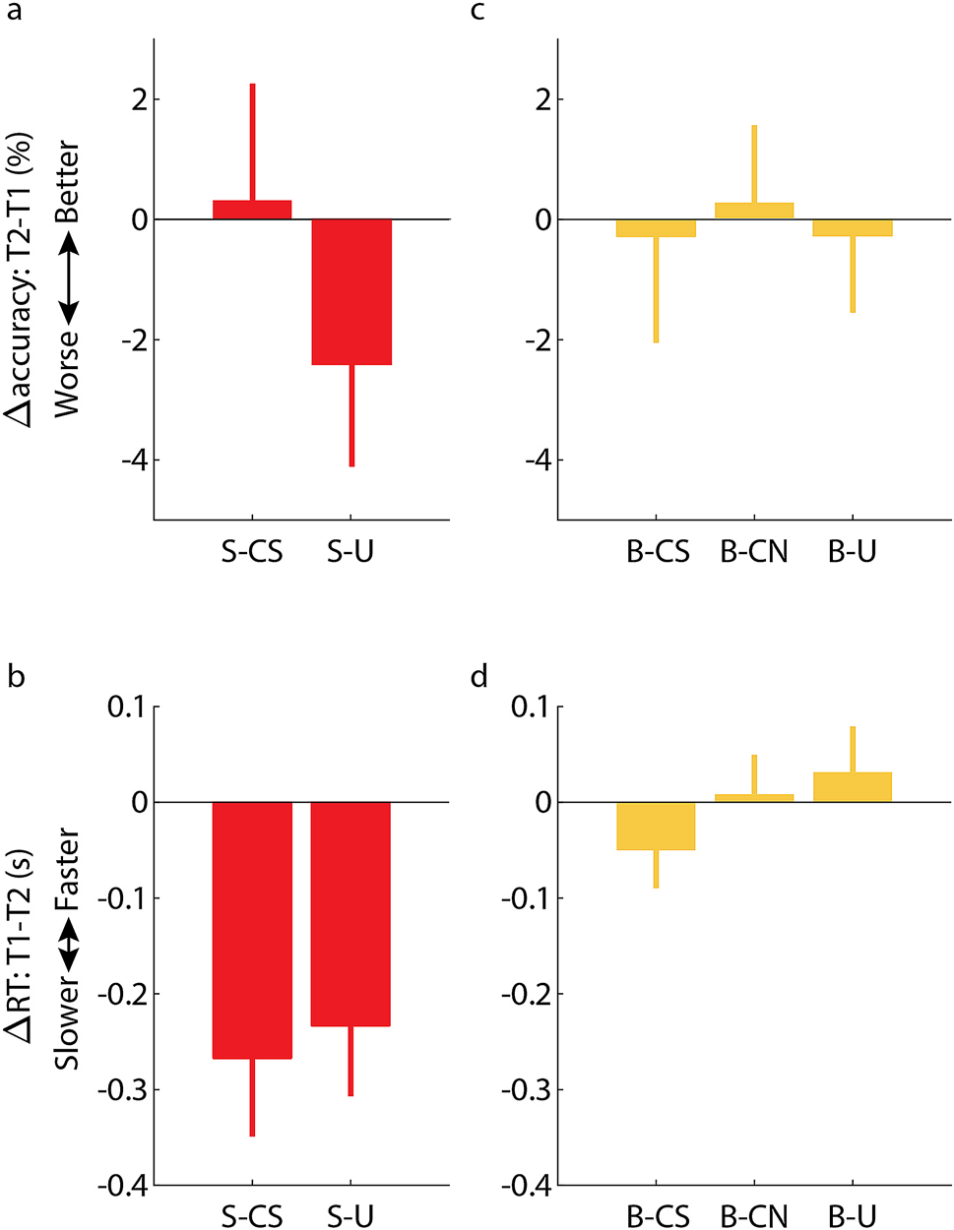
Reactivation during sleep did not have a suppressive effect. (**a**) Change in accuracy from T1 to T2 was similar for no-think pairs that were cued during sleep (S-CS; suppression–cued suppression) and those that were not (S-U; suppression–uncued). (**b**) A parallel analysis showed no differences in RT between the two conditions. (**c**) Change in accuracy was also similar when compared among the three baseline conditions, corresponding to pairs cued with the no-think sound (B-CS; baseline–cued suppression), pairs cued with a novel sound (B-CN; baseline–cued novel), and pairs that were uncued (B-U; baseline–uncued). (**d**) A parallel analysis was conducted for the change in RT. Values reflect coefficient estimates produced by the linear model; error bars denote standard error of the mean for coefficients.

To test the first hypothesis that cuing with the no-think sound during sleep would enhance suppression, we compared results from the S-CS set with the S-U set. These two conditions included identical procedures aside from sleep cuing, and there were no differences between T1 results for accuracy or RT (*t*(738) = 0.05, *p* = 0.96 and *t*(593) = 0.66, *p* = 0.51, respectively). Considering the T1-T2 change for both conditions, we found no effect of TMR on accuracy or RT (*t*(738) = − 1.19, *p* = 0.24, Fig. 3a and t(516) = 0.64, *p* = 0.52, Fig. 3b, respectively). To consider whether the data support the null hypotheses, we complemented these analyses with within-subject one-way paired t-tests (*t*(30) = 1.3, *p* = 0.9 for accuracy; *t*(30) =− 0.47, *p* = 0.68 for RT). The calculated Bayes factors showed strong evidence for the null hypothesis that TMR did not decrease memory accuracy (*BF*_*10*_ = 0.08) and moderate evidence for the null hypothesis that TMR did not increase RTs (*BF*_*10*_ = 0.14). Nonsignificant differences between the average changes hinted at a trend for better recall accuracy for the cued set relative to the uncued one. There was thus no evidence that TMR facilitated memory suppression.

To consider whether TMR can induce suppression de-novo during sleep, we compared results for two sets of baseline words (i.e., words that did not undergo the TNT manipulation). Some baseline words were preceded by a sound that signified suppression (B-CS) and others by a novel sound that was not presented during wake (B-CN). These two conditions were analyzed together with a third baseline condition with no cues during sleep (B-U). There was no evidence for differences among the three conditions in change in accuracy or RT (*F*(2,1109) = 0.06, *p* = 0.95, Fig. 3c and F(2,870) = 1.6, *p* = 0.2, Fig. 3d, respectively). Similar results were found with only the two cued conditions (B-CS and B-CN, both *p* > 0.21; *BF*_*10*_ < 0.24).

Finally, we considered the correlation between the number of times cues were presented to each participant during sleep (see Table 2) and the change in memory for the cued conditions. We tested whether the number of cues presented for each of the cued sets (S-CS, B-CS and B-NS) predicted change in performance (i.e., RTs and accuracy rates). No significant correlations were found for any of these conditions (*p* > 0.16). In conclusion, our results showed no evidence that TMR during sleep impaired memory.

### TMR improved memory for weakly encoded information

Given the many prior findings of memory improvement with TMR^19^, we conducted post-hoc analyses to test the hypothesis that TMR selectively improved memory. In a set of exploratory analyses, we considered that an improvement might have been produced selectively for weakly encoded memories (e.g.,^29^). Our task was not originally intended to manipulate encoding strength (see discussion of this limitation below). Nevertheless, we attempted to operationalize the concept of memory strength in our task using two complementary definitions, each with its own limitations and strengths.

First, we took advantage of the structure of our learning procedure, whereby participants are tested on all 86 word-pairs multiple times, up until at least 40% of them are recalled correctly. All pairs, regardless of whether they are remembered correctly, later undergo testing and TNT procedures. We therefore postulated that pairs that were never correctly recalled during training (Fig. 1b) were more weakly encoded than those that were recalled at least once. Although words were not recalled during training in the former case, they were presented to the participant at least twice during training, once during initial exposure (Fig. 1a) and one to three times as feedback during recall practice (Fig. 1b). We reasoned that these exposures during learning may have produced a sufficiently strong memory trace for TMR to enhance during sleep. Across participants, 58.06% ± 1.61% (SEM) of the words were recalled correctly at least once. The probability of successful recall was similar when compared among the three conditions that would later undergo the think-no-think manipulation (*F*(2,1113) = 0.15, *p* = 0.86) and among the three baseline conditions (*F*(2,1113) = 1.04, *p* = 0.35).

**Figure 4:**
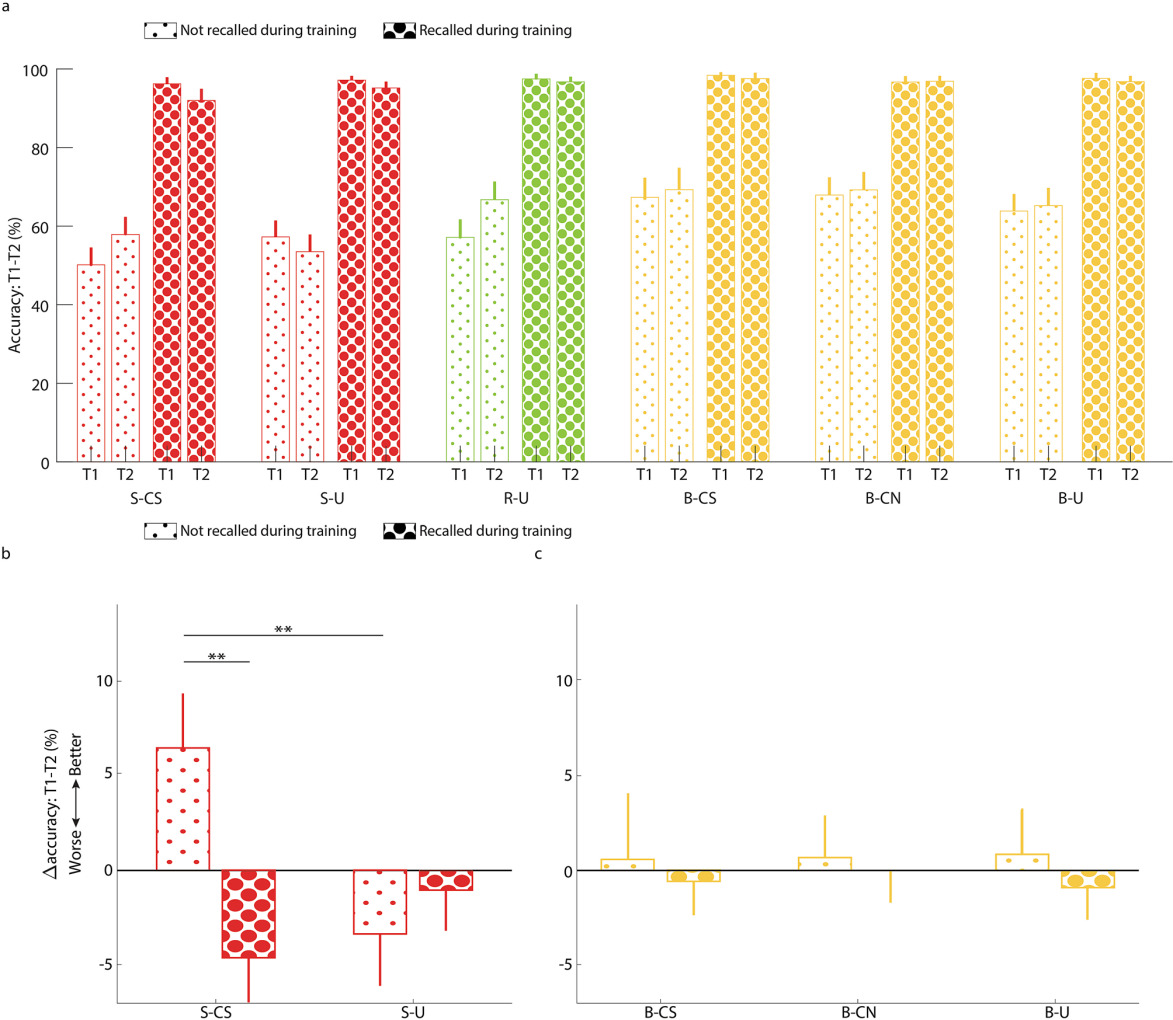
The benefit of reactivation for weakly encoded memories, as revealed by considering recall during training. (**a**) Recall accuracy in T1 and T2 as a function of condition and whether recall occurred during training (large dots) or did not occur during training (small dots). Error bars denote standard error of the mean between participants. (**b**) Changes in recall accuracy compared for the two suppression conditions, showing a memory improvement preferentially for the words that were not recalled during training. (**c**) A parallel analysis conducted for the three baseline conditions. Values in panels (**b**) and (**c**) reflect coefficient estimates produced by the linear model; error bars in panels (**b**) and (**c**) denote standard error of the mean for coefficients. **p < 0.01. *S-CS *suppression–cued suppression, *S-U* suppression–uncued, *R-U* response–uncued, *B-CS* baseline–cued suppression, *B-NS* baseline–cued novel, *B-U* baseline–uncued.

We compared the effects of TMR on the change in recall accuracy between T1 and T2, taking into account recall success during training (Fig. 4a). First, an analysis of only the two suppression conditions (S-CS, S-U) showed a significant positive effect of TMR (*F*(1,736) = 7.54, *p* < 0.01) and a significant interaction of TMR and training performance (*F*(1,736) = 7.73, *p* < 0.01; Fig. 4b). We also found a significant main effect of training performance (*F*(1,736) = 10.75, *p* < 0.01), but this effect may trivially reflect the tendency for bigger improvements with weakly encoded pairs than with well-learned pairs. The interaction effect was driven by a significantly greater TMR-enhancement for weakly encoded relative to strongly learned pairs (*t*(736) = 3.28, *p* < 0.01). This benefit was specific to the TMR condition, as reflected by a greater improvement with cues for weakly encoded words relative to strongly learned words (*t*(736) = 2.75, *p* < 0.01).

Conducting the same analysis on the baseline conditions did not yield significant effects (TMR: *F*(2,1106) = 0.003, *p* = 0.99; Training: *F*(1,1106) = 0.1, *p* = 0.76; interaction: *F*(2, 1106) = 0.04, *p* = 0.95; Fig. 4c). As discussed below, the beneficial effects of TMR in our task may have arisen from the presentation of the hint words more than from the instruction sounds. Accordingly, TMR effects for the B-CS and B-CN may have been similar even though instruction sounds differed. Yet, when these two baseline conditions were considered together and compared with B-U, the analysis again yielded nonsignificant results (*p* > 0.71 for all effects).

To establish that TMR differently affected weakly encoded memories in the baseline and suppressed conditions, we analyzed data from these four conditions together (i.e., S-SC and S-U; B-SC and B-U). This 2 × 2 × 2 analysis (suppression vs. baseline, cued vs. non-cued, weakly encoded vs. strongly encoded) yielded no main effects for any of these factors (*p* > 0.25). However, there was trend towards an interaction between cuing status and suppression/baseline, stemming from better performance measures for cued, suppressed items relative to non-cued ones (*F*(1,1474) = 3.29, *p* = 0.07). Importantly, a significant three-way interaction indicated that there was a benefit for weakly encoded items that were cued over non-cued items, but this benefit was limited to the suppression condition (*F*(1,1474) = 4.1, *p* < 0.05). All other interaction terms were not significant (*p* > 0.39). In conclusion, cuing benefited weakly encoded memories selectively for the weakly encoded suppressed pairs. Perhaps memories for words in the suppression condition differed from memories for words in the baseline condition in ways that influenced the outcome of TMR.

**Figure 5:**
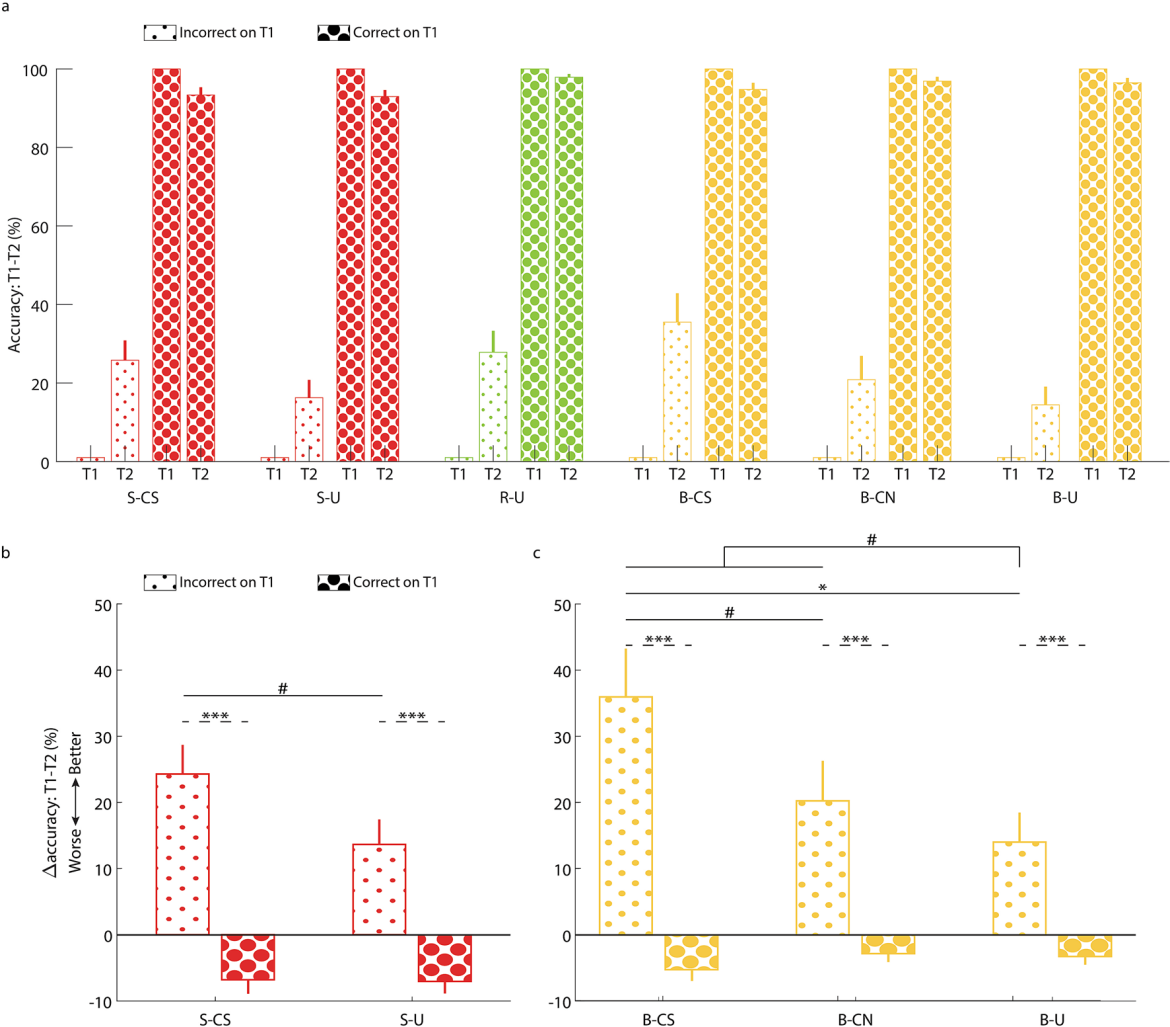
The benefit of reactivation for weakly encoded memories, as revealed by considering accuracy rates in T1. (**a**) Recall accuracy in T1 and T2 as a function of condition and whether recall occurred at T1 (large dots) or did not occur at T1 (small dots). Note that T1 results for all non-recalled conditions are at zero and the T1 results for all recalled conditions are at 100%, by definition. Error bars denote standard error of the mean between participants. (**b**) Changes in recall accuracy compared for the two suppression conditions, showing a trend toward memory improvement preferentially for the words that were not recalled in T1. There were also significant differences that were a byproduct of our segregation between items recalled in T1 and those that were not recalled. These differences are marked by dashed lines. (**c**) A parallel analysis conducted for the three baseline conditions showed a marginal difference between items that were not recalled at T1 in the B-CS condition and those of the B-CN condition; and a significance difference between items from the B-CS and B-U conditions that were not recalled in T1. Values in panels (**b**) and (**c**) reflect coefficient estimates produced by the linear model; error bars in panels (**b**) and (**c**) denote standard error of the mean for coefficients. ^#^*p* < 0.1, *p < 0.05; ***p < 0.001. *S-CS* suppression–cued suppression, *S-U *suppression–uncued, *R-U* response–uncued, *B-CS *baseline–cued suppression, *B-NS *baseline–cued novel, *B-U *baseline–uncued.

To complement this operationalization of weakly encoded memories, we used another measure to approximate encoding strength. The previous definition, based entirely on training data, may be contaminated by last-minute learning, in that some words not recalled during training may have been committed to memory following feedback, in which case some of the memories categorized as weak may have been strong. We therefore supplemented this analysis with another, this time distinguishing between pairs that were correctly recalled in T1 (i.e., the previously mentioned conditionalized group; Fig. 2c) and pairs that were not (Fig. 5a). This method is equivalent to previous analyses of per-item fates, whereby item memory gained, maintained, or lost over a delay are assessed separately, although that line of research did not commonly conceptualize these differences in terms of memory strength^28,30^. Although this operationalization of weakly encoded memories has higher validity than the one based on the training data, it is statistically less powerful. In total, less than 18% of pairs were not correctly recalled in T1, leaving a relatively small sample size per participant. However, our analytic approach (i.e., using linear mixed models) overcomes this limitation by pooling together trials across participants while accounting for between-subject variability.

As before, we first considered the effects of TMR on suppression by analyzing the S-CS and S-U conditions (Fig. 5b). We found a trend towards an effect of TMR (*F*(1,736) = 2.99, *p* = 0.08), driven by marginally greater improvements for the S-CS pairs that were not recalled in T1 (*t*(736) = 1.73, *p* = 0.08). The interaction effect between TMR and T1-recall was not significant (*F*(1,736) = 2.59, *p* = 0.11). We also revealed a main effect of T1-recall (*F*(1,736) = 44.25, *p* < 0.001). However, this effect is a byproduct of our choice of variables, since non-recalled items can only improve and recalled ones can only decline in a subsequent test.

Results for the baseline conditions followed similar lines. We found a trend towards a main effect among the B-CS, B-CN, and B-U conditions (*F*(2,1106) = 2.83, *p* = 0.06), a significant TMR by T1-recall effect (*F*(2,1106) = 3.57, *p* < 0.05) and a significant main effect of T1-recall (*F*(1,1106) = 35.37, *p* < 0.001; Fig. 5c). These effects were driven by a significant difference between the B-CS pairs and the B-U pairs that were incorrect in T1 (*t*(1106) = 2.38, *p* < 0.05), as well as a marginal difference between the B-CS pairs and the B-CN pairs that were incorrect in T1 (*t*(1106) = 1.73, *p* = 0.08). Comparing the pairs that were correct in T1 among conditions (e.g., B-CS vs. B-CN; B-CS vs. B-U) did not yield any significant differences (*p* > 0.24). Pooling the B-CS and B-CN conditions together, as described above, produced similar results: a trend towards a main effect of cuing (*F*(1,1108) = 3.63, *p* = 0.06), a significant interaction (*F*(1,1108) = 3.86, *p* < 0.05) and a trend towards higher improvement for the cued condition relative to the non-cued condition, when considering pairs that were incorrect in T1 (*t*(1108) = 1.91, *p* = 0.06).

Finally, we considered the effects of the suppression sound on both the baseline and suppression conditions’ word-pairs (i.e., S-SC and S-U; B-SC and B-U). This 2 × 2 × 2 analysis (suppression vs. baseline, cued vs. non-cued, correct vs. incorrect in T1) yielded three significant effects: a main effect of T1 results, which is a byproduct of our choice of variables (*F*(1,1474) = 14.67, *p* < 0.001); a main effect of cuing, explained by larger benefits for cued items (*F*(1,1474) = 5.82, *p* < 0.05); and an interaction between these two variables, whereby larger benefits are accrued by cued items that were incorrect in T1, regardless of condition (*F*(1,1474) = 7.07, *p* < 0.01). All other effects, including any differences between the baseline and suppression conditions, were not significant (*p* > 0.25). These results show that weakly encoded memories benefit from TMR regardless of whether they belong to baseline of suppression groups.

Our analyses for weakly encoded items intentionally focused on accuracy rates and not response times. The reasoning behind this decision is that RT data were necessarily restricted to trials with correct responses, given that most incorrect responses were omissions. Therefore, TMR-related differences in RTs could not be quantified effectively for weakly encoded pairs.

## Discussion

Our study was designed to test whether presentation of a suppression-related instructional sound during sleep in conjunction with memory reactivation would impair recall upon awakening. We used a variant of the established TNT paradigm and presented a subset of the no-think hint words during sleep. The impact of the TNT protocol on memory retrieval was as expected, decreasing recall for no-think word-pairs compared to baseline and think word-pairs in the conditionalized dataset (i.e., for word-pairs correctly recalled before the TNT manipulation). However, no-think cues during sleep did not boost memory suppression. Our second hypothesis, that cues can be used to suppress memories de-novo during sleep, was also not supported. Rather, post-hoc analyses revealed a memory benefit attributable to memory reactivation during sleep. This benefit was restricted to word-pairs that, before sleep, were weakly encoded, either in the baseline condition or in the no-think condition. Accordingly, we conclude that TMR preferentially strengthened weakly encoded memories. However, as discussed below, our experimental design was not optimized for testing this hypothesis, so these results merit some caution.

Our task varied from typical TNT designs in several respects: sounds were used to convey think and no-think instructions instead of visual cues; microphone recordings were used for the recall test; a relatively large set of word-pairs was used; and the first post-TNT test (T2) occurred after a relatively long break, which included a 90-min nap opportunity. Nonetheless, our results showed an effect of the TNT manipulation (Fig. 2) similar to that found in previous studies examining memory immediately after the TNT phase for conditionalized datasets (e.g.,^6,7^). Moreover, our results also demonstrate an equivalent effect when considering response times for correct responses rather than accuracy rates. Only two previous studies have reported the effects of a TNT manipulation on RTs^31,32^. RTs, which reflect memory accessibility, may be more sensitive to nuanced aspects of memory retrieval relative to the binary measure of accuracy commonly used in TNT designs. For example, our results showed that RTs for the suppressed condition remained slower than other conditions following a night of sleep (i.e., in T3; Fig. 2e,f), whereas the equivalent effects on accuracy were no longer significant (see below). Further research is needed to establish the validity of RT as a measure for TNT-related memory effects, potentially expanding the available toolbox for investigating memory control.

Our findings that TNT impacted memory retrieval in the conditionalized dataset following a delay of several hours (Fig. 2) stand in contrast with a recent study in which suppression-induced forgetting dissipated hours after the manipulation (regardless of whether this time was spent asleep^17^). These conflicting accounts may suggest that wake incubation counteracts memory control effects while sleep does not: the delay period used by Davidson and colleagues^17^ always included at least 1.5 h of wake, whereas our delay period consisted primarily of sleep. Alternatively, the null results presented in that study may be due to the specifics of their manipulation or to power concerns. Indeed, a different study showed evidence for a sustained difference between the think and no-think conditions over a 2.5-h period, regardless of whether or not participants napped during this delay (the study did not include a baseline condition)^16^.

Our study also examined memory for word-pairs on the day after the learning and TNT stages. Previous studies examining the persistence of the memory effects of the TNT manipulation over days have yielded conflicting results. Whereas Hotta and Kawaguchi^13^ found sustained effects of the manipulation on memory after 24 h, other studies showed no suppression-induced forgetting after a single night of sleep^15^ or after longer delays^11,12,33^. One difference between these studies that may partially explain these discrepancies concerns the strategy used by participants. In general, suppression-induced forgetting effects seem to last longer when participants use thought-substitution strategies (i.e., replacing the target word with another thought during the no-think phase) instead of thought-suppression strategies^12,13^. With regard to the duration of the effects of our TNT manipulation, our results are not clear cut: on the one hand, we found that the time of test (post-nap or on the next day) did not interact with TNT condition, indicating that the effects of the manipulation are not impacted by the long delay. This conclusion was also supported by the finding that suppression-induced forgetting, as reflected by slower RTs, was found both in T2 and T3. On the other hand, a planned analysis of T3 accuracy results did not show any effect of the TNT manipulation. Additionally, a significant interaction between time of test and condition was observed using a different analytic approach (i.e., proportional and not absolute accuracy scores; see Supplementary Analyses). Importantly, the participants were tested in both T2 and T3, and retrieval for the former test may have contaminated subsequent retrieval for the latter (i.e., a testing effect).

The purpose of our study was to consider the effects of targeted reactivation on memory suppression. Two previous studies have successfully used TMR to enhance memory suppression using cues associated with suppression in the context of directed forgetting tasks (in which participants are instructed not to commit recently presented information to memory^20,21^). The mechanism enabling this suppressive effect is unknown, but it may involve a reactivation of the suppressive context conjointly with a specific memory, thereby consolidating an inhibitory memory trace which counteracts its subsequent expression. These two studies differ in several respects: Simon and colleagues^20^ presented the suppressive memory cue in temporal proximity with a previously unrelated memory, effectively creating suppression de-novo during sleep; Schechtman and colleagues^21^ associated a single sound with both the suppressive action and a set of individual, previously suppressed memories, thereby enhancing previously established suppression during sleep.

Our failure to extend this literature to include TNT-induced memory control effects may have resulted from several factors. First, our study may have had insufficient power to reveal the contribution of TMR to memory suppression. However, this possibility seems unlikely given that we revealed an effect in the opposite direction for weakly encoded memories. It could still be that insufficient power masked a possible detrimental effect of TMR on stronger memories. Alternatively, it could be that cuing during sleep in our design induced both suppression and enhancement of memories, and these two effects nullified each other for stronger memories, but not weaker ones. Second, TMR may be ill-suited for enhancing suppression. This possibility is supported by null effects observed in studies exploring sleep’s role in memory suppression^14–17^, but results showing that suppression-induced forgetting can be achieved using unconscious exposure to suppression cues still hint at a potential to induce or enhance suppression during sleep^22^.

Finally, another possible explanation is that specific aspects of our protocol prevented enhancement of suppression. Because the suppression sound was paired with hint words during the TNT stage before sleep, this sound may have been linked with the learning context in a manner that competed with its link to the suppression context. If so, reactivation caused by the suppression sound during sleep may have preferentially acted to reinforce memory associations rather than weakening them. However, the protocol repeatedly cemented the association between the suppression sound and the TNT instructions.

Even granting this assumption that the suppression sound remained tightly and uniquely linked with the appropriate instructions, the cues during sleep may not have engaged suppression as intended. The consequences of processing two consecutive auditory stimuli during sleep are largely unknown with respect to how they may interact with each other, and particularly whether a second sound will disrupt processing provoked by the first sound. In our design, 1-s suppression cues were followed immediately by the hint word of the to-be-suppressed pair. The same timing was used both in wake and in sleep. Timing of suppression sound and hint word presentation was carefully chosen to balance two considerations: (a) they should not be too far apart, so that their conjoint reactivation can signal suppression of the link from the hint word to the target word; and (b) they should not be too close, so as to avoid interference. In previous studies, two sounds in close temporal proximity during sleep were found to block memory benefits of reactivation^34,35^. Therefore, we decided to concatenate the sound and subsequent word to try to avoid detrimental interference by producing an elongated single stimulus. However, the 1-s interval between sound onsets may not have been optimal. If the suppression sound was not processed to the point of reactivating a suppression context prior to activating the meaning of the hint word, any suppression may have been nullified. By this scenario, concatenation of the two sounds may essentially have been equivalent to presenting the hint word alone, which would explain the unexpected memory facilitation. To overcome this potential interference, future studies may try to utilize different modalities; the suppression cue could be an odor that is coincident with a spoken word.

Despite the absence of suppression effects following the sleep manipulation, the results obtained from our post-hoc analyses provided insights regarding the consequences of memory reactivation. Previous studies have shown that sleep (and wake rest) preferentially benefits weakly encoded memories (e.g.,^36–39^) (but see^14,40^). Whereas strongly encoded information may be more easily retrieved regardless of sleep, it seems that weaker memories may be more reliant on sleep-related consolidation to rescue them from subsequent forgetting. More specifically, it seems that TMR often benefits these weakly encoded memories more than others, suggesting that the degree to which memories respond to TMR is indicative of their general susceptibility for sleep-related consolidation^29,41^. Our results provide further evidence supporting the role of sleep, and specifically memory reactivation, in the consolidation of weakly encoded memories. We operationalized memory strength in two ways: by considering correct responses during training (Fig. 4) and by considering whether pairs were correctly remembered in the final pre-sleep test (Fig. 5). This last analysis is akin to those presented in previous studies which examined per-item gains and maintenance over a period of sleep^28,30^. Both of our analyses suggest that cuing during sleep rescues memories that were more weakly encoded from forgetting, whereas strongly encoded pairs were maintained regardless of cuing. Similar cuing-related gains were recently revealed in a language-learning task, although results indicate that in this study, unlike our own, cuing enhanced memory maintenance as well^30^.

Taken together, this evidence demonstrates that even extremely weak memory traces—those that do not cross the threshold of explicit recall before sleep—may be amenable to benefit from reactivation during sleep. In the present case, these weak memories were presumably strong enough to allow a benefit to be obtained using TMR. One limitation of our study is that our measure of memory strength was post-hoc and uncontrolled. It was based entirely on pre-sleep variability in memory performance, as measured for each participant. Although our study did use a controlled method to impact memory strength, suppression did not, by itself, interact with cuing (i.e., accuracy for cued suppressed items did not significantly improve during sleep relative to non-cued suppressed items; Fig. 3a). Only when accounting for pre-sleep memory levels does a cuing benefit emerge, limited to weakly encoded items.

Another limitation stemming from the post-hoc nature of our analysis concerns the uncontrolled effects of the TNT manipulation, especially when considering items that were not remembered in T1 (Fig. 5). During the TNT stage, participants were instructed to suppress some of these items, but it is unclear how one could suppress unretrievable memories and what the impact of such an action would be. This is one example of how our design introduces factors that are unrelated to memory strength and consequently complicates interpretation. Nevertheless, our results for items that were not remembered at T1 show that their benefit from TMR is not dependent on whether they were to be suppressed or part of the baseline word-pair group (Fig. 5). In other words, memory encoding strength predicted the benefit from TMR, and this effect was not significantly impacted by whether or not the item was part of a suppressed word-pair group. Therefore, despite the noted limitations, our results are consistent with the possibility that weakly encoded memories benefit from TMR.

A more general methodological concern is that separating strongly and weakly encoded memories may raise concerns about ceiling effects that may artificially limit our ability to observe sleep-related benefits for strongly-learned items. Indeed, we concede that strongly encoded memories have less to gain from reactivation during sleep. However, we believe these concerns do not threaten the validity of our results. First, declarative memories commonly decay over time, including during sleep. Our within-subject approach (i.e., comparing cued vs. non-cued with similar pre-sleep accuracy levels) allows us to compare differences in per-item forgetting between cued and non-cued pairs which could only be a product of cuing and are not hampered by ceiling effects. Second, we suggest that at-ceiling memories are not a limitation in experimental design, but rather an ecological key feature of strong memories. However, revealing subtle effects of sleep on memory may be hindered by combining malleable weak memories with over-trained at-ceiling memories, which tend to remain strong and have little to gain from reactivation during sleep. We therefore argue that at-ceiling memories should be considered separately from other memories to avoid Type II errors.

Despite growing appreciation of the beneficial nature of sleep for memory consolidation, the boundary conditions and neurocognitive mechanisms driving these benefits are poorly understood. Furthermore, a wealth of possible strategies for hacking into sleep could improve life for healthy and clinical populations—if we can understand the relevant neurocognitive mechanisms^10^. In this study, we sought to develop a method for the enhancement of memory suppression, which commonly comes into play for the inhibition of nonadaptive memories^5^. Instead, our results demonstrate the contribution of sleep to weakly encoded memories, which seems to be a fundamental property of sleep^42^. Our results thus lay the groundwork for an improved understanding of sleep-related consolidation and the potential to harness it for the better.

## Supplementary Information

Supplementary Information.
