# Supplementary material for: Sleep reactivation did not boost suppression-induced forgetting
Source: Sci Rep. 2021 Jan 14;11:1383. doi: 10.1038/s41598-020-80671-w (PMC7809483; doi:10.1038/s41598-020-80671-w)
Supplement: Supplementary file 1 — Supplementary Information. [file 41598_2020_80671_MOESM1_ESM.docx]

Title: **Sleep reactivation did not boost suppression-induced forgetting**

Eitan Schechtman, Anna Lampe, Brianna J Wilson, Eunbi Kwon, Michael C Anderson, Ken A Paller

Supplementary Materials include:

Supplementary Table 1

Supplementary Table 2

Supplementary Table 3

Supplementary Analyses

**Supplementary Table 1** – Word pairs used in the experiment. Each of the six sets was assigned to one of the experimental conditions

| **Set 1** | |  | **Set 2** | |  | **Set 3** | |  | **Set 4** | |
| --- | --- | --- | --- | --- | --- | --- | --- | --- | --- | --- |
| **Hint** | **Target** |  | **Hint** | **Target** |  | **Hint** | **Target** |  | **Hint** | **Target** |
| Accident | Snow |  | Booth | Dial |  | Ace | Symbol |  | Surface | Foam |
| Loan | Poverty |  | Lice | Scalp |  | Cent | Beggar |  | Vitamin | Lemon |
| Waffle | Maple |  | Mixture | Jar |  | Hive | Hexagon |  | Journey | Pants |
| Soil | Tomato |  | Abdomen | Thigh |  | Napkin | Fork |  | Stumble | Clown |
| Milk | Skim |  | Pipe | Wrench |  | Picnic | Hill |  | Fossil | Dinosaur |
| Slab | Granite |  | Tape | Radio |  | Crumb | Toaster |  | Bat | Dirt |
| Brake | Anchor |  | Crack | Lobster |  | Vault | Brass |  | Candle | Wine |
| Lawn | Beef |  | Lake | Summer |  | Coast | Thunder |  | Tree | Life |
| Needle | Doctor |  | Antler | Knife |  | Screen | Snack |  | Plane | Cloud |
| Lens | Physics |  | Helm | Opera |  | Luggage | Leather |  | Dough | Salt |
| Rear | Traffic |  | Museum | Clay |  | Decay | Carbon |  | Instant | Rate |
| Unit | Gravity |  | Wood | Termite |  | Pencil | Sticker |  | Society | Youth |
|  |  |  |  |  |  |  |  |  |  |  |
| **Set 5** | |  | **Set 6** | |  |  |  |  | **Filler pairs** | |
| **Hint** | **Target** |  | **Hint** | **Target** |  |  |  |  | **Hint** | **Target** |
| Breath | Tickle |  | Insight | Art |  |  |  |  | Bond | Wedding |
| Crow | Ascent |  | Pet | Mouse |  |  |  |  | Cartoon | Hero |
| Jogger | Collie |  | Relax | Bed |  |  |  |  | Chalk | Book |
| Servant | Queen |  | Broom | House |  |  |  |  | Cigar | Vice |
| Latch | Cage |  | Cradle | Parent |  |  |  |  | College | Certificate |
| Liberty | Eagle |  | Business | Desk |  |  |  |  | Ego | Shallow |
| Surprise | Snake |  | Glow | Ghost |  |  |  |  | Jaw | Gum |
| Diet | Cream |  | Aroma | Plant |  |  |  |  | Lint | Curtain |
| Hug | Rose |  | Knuckle | Ring |  |  |  |  | Nail | Picture |
| Steam | Train |  | Decision | Judge |  |  |  |  | Pump | Oil |
| Nightclub | Lipstick |  | Metal | Staple |  |  |  |  | Scratch | Mosquito |
| Plum | Core |  | Leap | Ballet |  |  |  |  | Toll | Bridge |
|  |  |  |  |  |  |  |  |  | Type | Paper |
|  |  |  |  |  |  |  |  |  | Wisdom | Monk |

**Supplementary Table 2** – Responses to the questionnaire intended to verify that the TNT instructions were understood and implemented. The same questionnaire was filled out twice – after the first TNT practice round, and after three of the six TNT blocks

| Question | Range | During training  Mean ± SD | | | After TNT block #3  Mean ± SD | | |
| --- | --- | --- | --- | --- | --- | --- | --- |
| 1. For the Hint words presented with the “THINK” sounds, how often did you try to think of the associated RESPONSE word as fast as possible? | 0 – Never  4 – Always | 3.52 | ± | 0.85 | 3.77 | ± | 0.43 |
| 2. When Hint words were presented with “NO THINK” sounds, how much time did you spend looking at the Hint word, without shifting your eyes OR attention to something else? | 0 – Didn’t look at all  4 – Looked entire time | 2.94 | ± | 1.06 | 3.47 | ± | 0.62 |
| 3. For “NO THINK” trials, how often did you read and understand the Hint word? | 0 – Never  4 – Always | 3.87 | ± | 0.43 | 3.9 | ± | 0.3 |
| 4. How often were you able to not think about the Response word that went with the words presented with “NO THINK” sounds? | 0 – Never  4 – Always | 2.19 | ± | 0.83 | 2.85 | ± | 0.57 |
| 5. How often did you actively push the Response word out of mind if it did come to mind? | 0 – Never  4 – Always | 3.29 | ± | 1.07 | 3.67 | ± | 0.66 |
| 6. How often did you think about something else to distract yourself, such as another image, word, or idea? | 0 – Never  4 – Always | 1.32 | ± | 1.35 | 0.48 | ± | 0.57 |
| 7. Did you ever intentionally think about the Response word “just for a second” to see if you still knew it? | 0 – Never  4 – Always | 0.65 | ± | 0.71 | 0.19 | ± | 0.4 |
| 8. How often did you think about the Response word after the Hint word went off the screen? | 0 – Never  4 – Always | 0.61 | ± | 0.84 | 0.52 | ± | 0.51 |

**Supplementary Table 3** – Responses to the post-task questionnaire

| Question | Range | Mean ± SD | | |
| --- | --- | --- | --- | --- |
| 1. For the trials in which you were trying to avoid thinking of the RESPONSE word, how did your ability to avoid thinking of the RESPONSE word change over the number of times you saw the word? | 0 – Got easier  4 – Got harder | 1.1 | ± | 0.92 |
| 2. When I saw the heard a “NO THINK” sound with a HINT word, I quickly checked to see if I remembered the response word. | 0 – Never  4 – Very frequently | 0.87 | ± | 0.68 |
| 3. After the “NO THINK” hint words went off the screen, I checked to see if I still remembered the response word. | 0 – Never  4 – Very frequently | 0.63 | ± | 0.76 |
| 4. When I saw the “NO THINK” hint words, I thought about the response that went with it to improve my memory for that word pair. | 0 – Never  4 – Very frequently | 0.37 | ± | 0.61 |
| 5. I stared intently at the Hint word presented with a “NO THINK” sound. To divert my mind from thinking about the response word, I focused on the individual letters of the word, or the words overall visual appearance | 0 – Never  4 – Always | 3.07 | ± | 0.91 |
| 6. I repeated the Hint word to myself. To divert my mind from thinking of the response word, I said the Hint word to myself again and again to keep my mind occupied. | 0 – Never  4 – Always | 3.42 | ± | 0.62 |
| 7. I used The Hint word to Generate Related Word or Thought. To divert my mind, I used the Hint word to generate a related word or idea that is associated to the word. | 0 – Never  4 – Always | 1.07 | ± | 0.83 |
| 8. I used the Hint word to Generate Personal Memory. To divert my mind, I used the Hint word to generate a memory of an event from my life that was related to the word. | 0 – Never  4 – Always | 0.43 | ± | 0.68 |
| 9. I used the Hint Word to Generate a Sound. To divert my mind, I thought of a sound or music related to the hint word. | 0 – Never  4 – Always | 0.33 | ± | 0.55 |
| 10. I stared Blankly at the Hint word and Kept mind Clear. Although I kept looking at the Hint word, I didn’t analyze how it appeared; I just cleared my mind and thought of nothing. | 0 – Never  4 – Always | 1.93 | ± | 0.87 |
| 11. I refocused my attention to another sensation. When the Hint word came up, I kept looking at the word, but I shifted my attention to sounds in the room or outside; or I thought about another sensation (e.g., how comfortable your posture is, a taste in your mouth, etc.). | 0 – Never  4 – Always | 1.03 | ± | 1.13 |
| 12. I refocused my attention on other unrelated thoughts. When the Hint word came up, I kept looking at the word, but I just “checked out” and thought about things unrelated to the experiment—e.g., what happened last night, what I will do later today, or anything else. | 0 – Never  4 – Always | 0.83 | ± | 0.87 |
| 13. I played Word Games with the Hint Word. When the Hint word came up, I searched for other words that could be made from the letters. | 0 – Never  4 – Always | 0.23 | ± | 0.57 |
| 14. I refocused my attention on a “distracting task”. For instance, you might have counted backwards by 3s or some other task to keep your mind occupied. | 0 – Never  4 – Always | 0.3 | ± | 0.65 |
| 15. I diverted my attention from the Hint word. Although I kept my eyes on the Hint word, I shifted my attention covertly to a different location, so I could avoid looking at the reminder. | 0 – Never  4 – Always | 0.77 | ± | 0.82 |
| 16. I diverted my eyes. Same as above, but when the Hint word came up, I simply moved my eyes away so I didn’t have to look at it. | 0 – Never  4 – Always | 0.83 | ± | 0.87 |
| 17. When I heard a “THINK” sound, I was able to associate the sound with thinking about the Response. | 0 – Never  4 – Always | 3.17 | ± | 0.95 |
| 18. When I heard a “NO THINK” sound, I was able to associate the sound with not thinking about the Response. | 0 – Never  4 – Always | 3.17 | ± | 0.95 |
| 19. In one phase of the experiment, we asked you to not think about the associated response word for HINT words presented with a “NO THINK” sound. During this phase, did you suspect that you would later be asked to recall the responses for these HINT words? In other words, did you anticipate some form of a final test? | 0 – No, I didn’t  4 – Yes, I did | 2.77 | ± | 1.3 |
| 20. Did you rehearse the words pairs, even unintentionally, between the first session and the second one? | 0 – No  1 – Yes | 0.03 | ± | 0.18 |

**Supplementary Analyses**

The analyses presented in the manuscript was conducted using mixed linear models. In order to allow direct comparisons between our results and those used in some other papers, we include here additional analyses of our data that do not rely on these models. Using these methods, we produce results which are qualitatively similar to those presented in the Results sections titled “Think-no-think procedures altered later memory retrieval” and “No evidence that TMR enhanced suppression”. One exception to this pattern of similarity is that this new analysis produced a significant interaction between delay (T2 vs. T3) and condition (S-U vs. R-U vs. B-U) in the non-cued sets, as explained below.

For this analysis, we first calculated the average T1 accuracy rates for each condition and each participant. T2 and T3 results (i.e., those obtained from later tests) were then divided by T1 results, setting the accuracy at T1 for each condition and participant to 100%. This was done both for the conditionalized dataset (i.e., taking into account only pairs that were correct at T1) and for the complete dataset. Data was then submitted to a set of repeated-measures ANOVAs (rmANOVA). Post-hoc comparisons were done using Tukey’s Honest Significant Difference.

**Think-no-think procedures altered later memory retrieval**

For the complete dataset:

- A 2 X 3 rmANOVA considering delay (T2 vs. T3) and condition (S-U vs. R-U vs. B-U) for the non-cued sets revealed a main effect of delay (*F*(1,29) = 5.42, *p* < 0.05; accuracy higher for T3). Additionally, the analysis revealed a main effect of condition (*F*(2,58) = 3.86, *p* < 0.05), with post-hoc comparisons revealing that this effect stemmed from memory for the S-U condition being worse than for the R-U condition (*p* < 0.05). Finally, the interaction between the two factor was also significant (*F*(2,58) = 4.45, *p* < 0.05). Post-hoc analyses showed that memory for the S-U condition was significantly worse than for the R-U condition in the T2 test (*p* < 0.05). All other comparisons were non-significant (*p* > 0.12).
- A 3-way rmANOVA considering T2 accuracy for the different conditions for the non-cued sets revealed a significant effect (*F*(2,60) = 6.1, *p* < 0.01). Post-hoc comparisons showed that memory for the S-U condition was significantly worse than for the R-U condition (*p* < 0.01). All other comparisons were non-significant (*p* > 0.09).
- A 3-way rmANOVA considering T3 accuracy for the different conditions for the non-cued sets revealed a marginal effect (*F*(2,58) = 2.52, *p* = 0.09). Post-hoc comparisons did not find any significant differences between the conditions (*p* > 0.14).

For the conditionalized dataset:

- A 2 X 3 rmANOVA considering delay (T2 vs. T3) and condition (S-U vs. R-U vs. B-U) for the non-cued sets revealed a trend towards a main effect of condition (*F*(2,58) = 2.99, *p* = 0.06), with post-hoc comparisons revealing that this effect stemmed from memory for the S-U condition being worse than for the R-U condition (*p* < 0.05). No main effect of delay was observed for this dataset (*F*(1,29) = 1.05, *p* = 0.31). Finally, the interaction between the two factor was significant (*F*(2,58) = 5.46, *p* < 0.01). Post-hoc analyses showed that memory for the S-U condition was significantly worse than for the R-U condition in the T2 test (*p* < 0.05). All other comparisons were non-significant (*p* > 0.09).
- A 3-way rmANOVA considering T2 accuracy for the different conditions for the non-cued sets revealed a significant effect (*F*(2,60) = 5.71, *p* < 0.01). Post-hoc comparisons showed that memory for the S-U condition was significantly worse than for the R-U condition (*p* < 0.05). All other comparisons were non-significant (*p* > 0.09).
- A 3-way rmANOVA considering T3 accuracy for the different conditions for the non-cued sets did not yield a significant effect (*F*(2,58) = 0.88, *p* = 0.42).

**No evidence that TMR enhanced suppression**

For the complete dataset:

- A 2-way rmANOVA considering cued vs. non-cued suppression conditions (S-CS vs. S-U) did not reveal an effect (*F*(1,30) = 2.78, *p* = 0.11). The non-significant trend is toward better memory in S-CS relative to the S-U condition. Note that this analysis is identical to conducting a paired t-test, but was presented in ANOVA form for consistency.
- A 3-way rmANOVA considering the three baseline conditions (B-CS vs. B-NS vs. B-U) did not reveal an effect (*F*(2,60) = 0.01, *p* = 0.99).

For the conditionalized dataset:

- A 2-way rmANOVA considering cued vs. non-cued suppression conditions (S-CS vs. S-U) did not reveal an effect (*F*(1,30) = 0.04, *p* = 0.84). Note that this analysis is identical to conducting a paired t-test, but was presented in ANOVA form for consistency.
- A 3-way rmANOVA considering the three baseline conditions (B-CS vs. B-NS vs. B-U) did not reveal an effect (*F*(2,60) = 0.77, *p* = 0.47).
